# Unraveling the Impact of miR-146a in Pulmonary Arterial Hypertension Pathophysiology and Right Ventricular Function

**DOI:** 10.3390/ijms25158054

**Published:** 2024-07-24

**Authors:** Joana Santos-Gomes, Pedro Mendes-Ferreira, Rui Adão, Carolina Maia-Rocha, Beatriz Rego, Manu Poels, Anaïs Saint-Martin Willer, Bastien Masson, Steeve Provencher, Sébastien Bonnet, David Montani, Frédéric Perros, Fabrice Antigny, Adelino F. Leite-Moreira, Carmen Brás-Silva

**Affiliations:** 1 Cardiovascular R&D Centre–UnIC@RISE, Department of Surgery and Physiology, Faculty of Medicine, University of Porto, 4200-319 Porto, Portugal; joanapxgomes@gmail.com (J.S.-G.); pedmendesferreira@gmail.com (P.M.-F.); ruimigueladao@gmail.com (R.A.); carolina_rocha86@msn.com (C.M.-R.); silvaregobeatriz@gmail.com (B.R.); manu.poels@student.uantwerpen.be (M.P.); amoreira@med.up.pt (A.F.L.-M.); 2 Paris-Porto Pulmonary Hypertension Collaborative Laboratory (3PH), UMR_S 999, INSERM, Université Paris-Saclay, 91190 Paris, France; frederic.perros@inserm.fr; 3 Department of Pharmacology and Toxicology, School of Medicine, Universidad Complutense de Madrid, 28040 Madrid, Spain; 4 CIBER Enfermedades Respiratorias (Ciberes), 28029 Madrid, Spain; 5 Instituto de Investigación Sanitaria Gregorio Marañón (IiSGM), 28007 Madrid, Spain; 6 Assistance Publique-Hôpitaux de Paris (AP-HP), Department of Respiratory and Intensive Care Medicine, Pulmonary Hypertension National Referral Center, Hôpital de Bicêtre, 94270 Le Kremlin-Bicêtre, France; anais.saint-martin@universite-paris-saclay.fr (A.S.-M.W.); bastienmasson999@gmail.com (B.M.); david.montani@aphp.fr (D.M.); fabrice.antigny@inserm.fr (F.A.); 7 Inserm UMR-S 999 «Pulmonary Hypertension: Pathophysiology and Novel Therapies», Hôpital Marie Lannelongue, 92350 Le Plessis-Robinson, France; 8 Pulmonary Hypertension Research Group, Centre de Recherche de l’Institut Universitaire de Cardiologie et de Pneumologie de Québec, Québec City, QC G1V 4G5, Canada; steeve.provencher@criucpq.ulaval.ca (S.P.); sebastien.bonnet@criucpq.ulaval.ca (S.B.); 9 Department of Medicine, Université Laval, Québec City, QC G1V 0A6, Canada; 10 Service de Pneumologie et Soins Intensifs Respiratoires, Centre de Référence de l’Hypertension Pulmonaire, Hôpital Bicêtre, 94270 Le Kremlin-Bicêtre, France; 11 CarMeN Laboratory, INSERM U1060, INRAE U1397, Université Claude Bernard Lyon 1, 69310 Pierre-Bénite, France; 12 Faculty of Nutrition and Food Sciences, University of Porto, 4099-002 Porto, Portugal

**Keywords:** miR-146a, pulmonary arterial hypertension (PAH), right ventricular (RV) hypertrophy, right ventricular (RV) failure

## Abstract

Pulmonary arterial hypertension (PAH) is a chronic disorder characterized by excessive pulmonary vascular remodeling, leading to elevated pulmonary vascular resistance and right ventricle (RV) overload and failure. MicroRNA-146a (miR-146a) promotes vascular smooth muscle cell proliferation and vascular neointimal hyperplasia, both hallmarks of PAH. This study aimed to investigate the effects of miR-146a through pharmacological or genetic inhibition on experimental PAH and RV pressure overload animal models. Additionally, we examined the overexpression of miR-146a on human pulmonary artery smooth muscle cells (hPASMCs). Here, we showed that miR-146a genic expression was increased in the lungs of patients with PAH and the plasma of monocrotaline (MCT) rats. Interestingly, genetic ablation of miR-146a improved RV hypertrophy and systolic pressures in Sugen 5415/hypoxia (SuHx) and pulmonary arterial banding (PAB) mice. Pharmacological inhibition of miR-146a improved RV remodeling in PAB-wild type mice and MCT rats, and enhanced exercise capacity in MCT rats. However, overexpression of miR-146a did not affect proliferation, migration, and apoptosis in control-hPASMCs. Our findings show that miR-146a may play a significant role in RV function and remodeling, representing a promising therapeutic target for RV hypertrophy and, consequently, PAH.

## 1. Introduction

Pulmonary arterial hypertension (PAH) is a severe and multifactorial disease, defined as an increase in mean pulmonary artery (PA) pressure (mPAP) > 20 mmHg (mPAP in healthy subjects at rest is 14.0 mmHg) [1], PA wedge pressure ≤ 15 mmHg, and pulmonary vascular resistance (PVR) of >2 Wood units at rest in the absence of other causes (e.g., conditions that can lead to increased mPAP, such as pulmonary hypertension (PH) associated with lung diseases, and chronic thromboembolic PH (CTEPH)) [2]. PAH is characterized by excessive pulmonary vascular remodeling, which results in an increase in pulmonary arterial obstruction and an increase in PVR, increasing the right ventricle (RV) afterload, RV hypertrophy, and eventually leading to death from RV failure (RVF), the major cause of PAH mortality and morbidity [3,4,5]. Currently, the available therapies for PAH are unable to cure the disease, merely diminishing the risk of short-term mortality and the clinical worsening of patients [6]. In the long term, the prognosis continues to be poor [7]; thus, new therapeutic approaches are required.

MicroRNAs (miRs) are small, single-stranded, non-coding RNA molecules that regulate gene expression post-transcriptionally by inhibiting translation or by degradation of the target RNA messenger through sequence-specific binding [8]. The ability of miRs to regulate diverse biological and cellular pathways makes their manipulation an attractive therapeutic approach. Potential intervention points include blocking or increasing miR production at the nuclear level, therapeutic miR replacement using miR mimics, inhibiting mature miR with anti-miRs, or interacting directly with target miRs [9]. Several studies have demonstrated1 that miRs are present in human serum and plasma. Their expression profile is altered in several cancers and could influence various cellular processes, including cell proliferation, differentiation, apoptosis, and metabolism [10]. MiRs are highly stable in fresh and stored plasma samples, making them promising blood-based biomarkers for diagnostic and prognostic purposes [11]. Circulating miRs have emerged as significant markers for cardiovascular diseases [12,13,14]. Although the pathogenesis of PAH is not fully understood, evidence shows that changes in the expression of miRs are involved in the initiation and development of PAH [15].

The microRNA-146 (miR-146) family consists of two highly conserved members: miR-146a and miR-146b. These largely homologous genes differ by only two nucleotides in their mature sequences at the 3′ end. In humans, miR-146a and miR-146b are encoded by genes located on separate chromosomes—chromosome 5 and chromosome 10, respectively [16,17]. MiR-146a is abundantly expressed in the heart [18], and its expression is upregulated in individuals with hypertrophic cardiomyopathy [13]. Studies have highlighted miR-146a’s essential role in the pathogenesis of various heart failure (HF) types and its involvement in cardiac hypertrophy [12,19,20]. MiR-146a is also involved in vascular systemic smooth muscle cell proliferation and neointima hyperplasia, both important hallmarks of PAH [21]. Circulating levels of miR-146a are increased in patients with coronary arterial disease [22], and miR-146a is overexpressed in the RV [23] and PA endothelial cells (PAECs) under hypoxic conditions [24]. In children with PAH, the overexpression of miR-146a in the circulation is correlated with an increase in PVR index [25]. Hence, miR-146a could be a potential therapeutic target for proliferative vascular diseases like PAH. Furthermore, miR-146a is crucial in immune responses and various inflammatory processes [26,27].

In our study, we describe the role of miR-146a in PAH and RV hypertrophy. We evaluate miR-146a expression in PAH patients and experimental models of PH. Moreover, we examine the effects of gene knockout (KO) and the pharmacological inhibition of miR-146a on PAH pathophysiology using different experimental models. Our findings demonstrate that inhibition of miR-146a protects the RV and cardiac dysfunction in vivo associated with the pathophysiology of PAH, suggesting its potential as a therapeutic target for PAH.

## 2. Results

### 2.1. MiR-146a Expression Is Increased in Human Lung PAH Tissues

**Figure 1:**
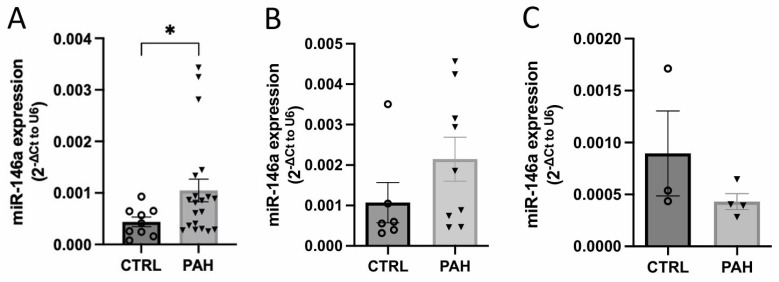
Expression of miR-146a in human lung samples from pulmonary arterial hypertension (PAH) (*n* = 20) and control (CTRL) (*n* = 10) patients (**A**). Expression of miR-146a in human pulmonary artery smooth muscle cells (hPASMCs) (*n* = 6–9 hPASMCs) (**B**) and human pulmonary artery endothelial cells (hPAECs) (*n* = 3–4 hPAECs) (**C**) from PAH and CTRL patients. Graph bars represent mean ± SEM. * *p* < 0.05.

Lung miR-146a expression in lung human samples was increased in PAH patients compared to control (CTRL) (Figure 1A). Furthermore, we correlated the expression of miR-146a with some parameters from patients such as systolic PA pressure (sPAP), mPAP, RV systolic pressure (RVSP), cardiac output (CO), and cardiac index (CI) (Figure S1). However, we did not see any correlation between miR-146a levels and these parameters. To understand why miR-146a expression is increased in the lung tissue of PAH patients, we evaluated the expression of miR-146a in human PA smooth muscle cells (hPASMCs) and hPAECs (Figure 1B and Figure 1C, respectively) from PAH and CTRL patients. However, there were no significant differences in miR-146a expression between CTRL and PAH patients.

### 2.2. Knockout (KO) Mice for miR-146a Show Improvements in RV in Response to SuHx

**Figure 2:**
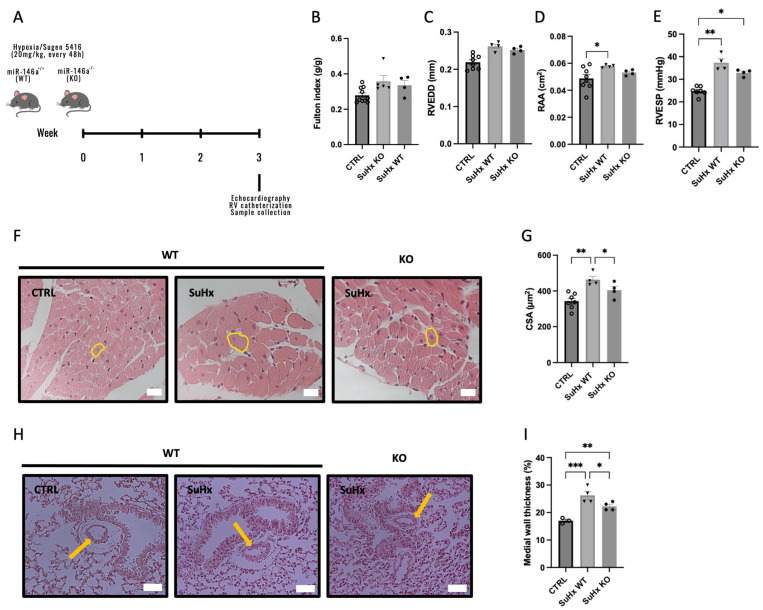
Wild-type (WT) and miR-146a knockout (KO) mice were exposed to 3 weeks of chronic hypoxia with weekly administrations of Sugen 5416 (SuHx) (**A**). Right ventricle (RV) hypertrophy, measured by the Fulton index, was not changed between experimental groups (**B**). Right heart dilation, measured by the RV end-diastolic dimension (RVEDD) (**C**) and right atria area (RAA) (**D**), presented an increase in WT mice submitted to SuHx. RV end-systolic pressure (RVESP) was increased in WT mice submitted to SuHx (**E**). Cardiomyocyte hypertrophy, measured by cardiomyocyte cross-sectional area (CSA): representative cross-sections of cardiomyocytes (a yellow circle in representative cardiomyocytes) (**F**) and the quantitative analysis of cardiomyocyte CSA (**G**). Representative pulmonary arterioles in the lung sections of mice (a yellow arrow in representative pulmonary arterioles) (**H**) and the quantitative analysis of medial wall thicknesses (measured by inner diameter/(inner diameter + outside diameter) of pulmonary arterioles) (**I**). Scale bars represent 20 and 50 μm in panels (**F**,**H**), respectively. Graph bars represent mean ± SEM for 3–10 mice. * *p* < 0.05, ** *p* < 0.005, *** *p* < 0.0005.

To determine the effects of miR-146a in the pathogenesis of PAH, we exposed miR-146a deficient mice to Sugen/hypoxia (SuHx). After 3 weeks submitted to hypoxia, the animals were euthanized (Figure 2A). As expected, WT mice, after 3 weeks submitted to SuHx (SuHx-WT), developed right atria (RA) dilation, measured by the RA area (RAA) (0.058 vs. 0.049 cm^2^) (Figure 2D), increased RVSP (37.35 vs. 24.76 mmHg) (Figure 2E), and increased RV cardiomyocyte cross-sectional area (CSA) (463.32 vs. 342.57 µm^2^) compared to CTRL (Figure 2F,G). However, no differences were observed in the Fulton index (expressing the degree of RV hypertrophy (Figure 2B)) and in RV end-diastolic diameter (RVEDD) (Figure 2C) between all experimental groups (CTRL, SuHx-WT, and SuHx-KO).

KO mice for miR-146a exposed to SuHx showed an increase in RVSP compared to WT mice (32.91 vs. 24.76 mmHg); however, it was not as pronounced as the increase in pressures in WT mice exposed to SuHx (Figure 2D). Regarding the dimensions of the RV, there were no differences between the WT and KO mice exposed to SuHx, neither in the diameter of the RV nor in the RAA (0.26 vs. 0.25 mm and 0.058 vs. 0.053 cm^2^, respectively; Figure 2C and Figure 2D, respectively). SuHx-KO mice had improved cardiomyocyte CSA compared to SuHx-WT mice (404.2 vs. 463.32 µm^2^) (Figure 2F,G). Regarding the thickness of the medial wall of PAs, mice submitted to SuHx presented an increase in the thickness compared with CTRL mice (26.25 and 22.25 vs. 17%) (Figure 2H,I). However, SuHx-KO mice showed improvements in the pulmonary wall thickness, compared to SuHx-WT mice (22.25 vs. 26.25%, respectively) (Figure 2H,I).

### 2.3. Knockout (KO) Mice for miR-146a Show Decreased RV Remodeling in Response to Pressure Overload

**Figure 3:**
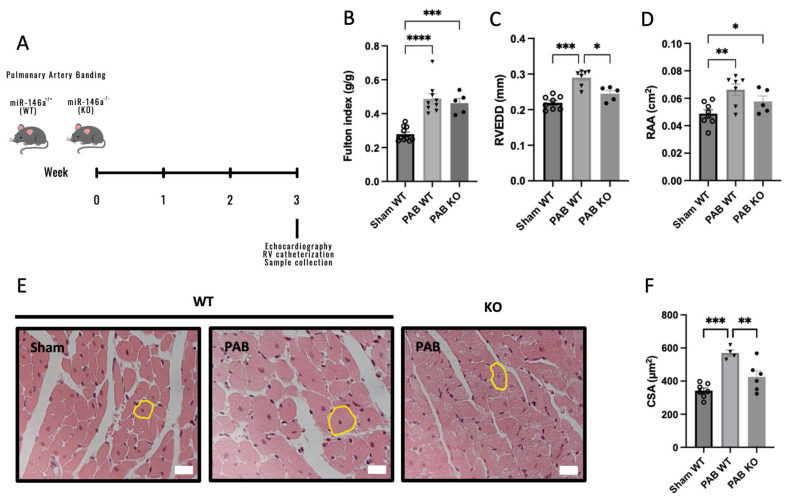
Wild-type (WT) and miR-146a knockout (KO) mice were exposed to 3 weeks of pulmonary artery banding (PAB) (**A**). Right ventricle (RV) hypertrophy, measured by the Fulton index, was increased in WT and KO mice submitted to PAB (**B**) compared to Sham WT mice, but the increase was more accentuated in the PAB WT group than in the PAB KO group. Right heart dilation, measured by the RV end-diastolic dimension (RVEDD) (**C**) and right atria area (RAA) (**D**), was also increased in WT and KO mice submitted to PAB, but was more accentuated in the PAB WT group. Cardiomyocyte hypertrophy, measured by cardiomyocyte cross-sectional area (CSA): representative cross-sections of cardiomyocytes (a yellow circle in representative cardiomyocytes) (**E**) and the quantitative analysis of cardiomyocyte CSA (**F**). Scale bar represents 20 μm in panel (**E**). Graph bars represent mean ± SEM for 4–10 mice. * *p* < 0.05; ** *p* < 0.005; *** *p* < 0.0005; **** *p* < 0.0001.

Afterward, we analyzed the consequence of genetic ablation of miR-146a in RV remodeling independently of pulmonary vascular changes-induced pulmonary artery banding (PAB) (Figure 3A) [28] to determine whether miR-146a inhibition could benefit RV function. RV hypertrophy (measured by Fulton index) (Figure 3B), together with RV dilation (measured by RVEDD) and RAA, increased after PAB in both genotypes, were compared to Sham-WT mice (Figure 3C and Figure 3D, respectively). However, PAB-WT mice showed an increase in RV hypertrophy compared to Sham-WT mice (0.49 vs. 0.28 g/g) and PAB-KO mice (0.49 vs. 0.46 g/g) (Figure 3B).

Regarding the RV dimensions, no significant differences were observed between Sham-WT and PAB-KO mice (0.22 vs. 0.24 mm), but we found an increase in RV dimensions in PAB-WT mice compared with PAB-KO mice (0.29 vs. 0.24 mm) (Figure 3C). In addition, we found an increase in RAA in PAB-WT and PAB-KO mice compared to Sham-WT mice (0.066 and 0.058 vs. 0.049 cm^2^, respectively). However, this increase in PAB-WT mice was higher than in PAB-KO mice (Figure 3D). Regarding RV cardiomyocyte CSA, we found an improvement in PAB-KO mice compared with PAB-WT mice (425.57 vs. 569.58 µm^2^), and similar levels of RV cardiomyocyte hypertrophy between Sham-WT mice and PAB-KO mice (342.57 vs. 425.57 µm^2^; Figure 3E,F).

### 2.4. MiR-146a Inhibition Results in Decreased RV Remodeling in Response to Pressure Overload

**Figure 4:**
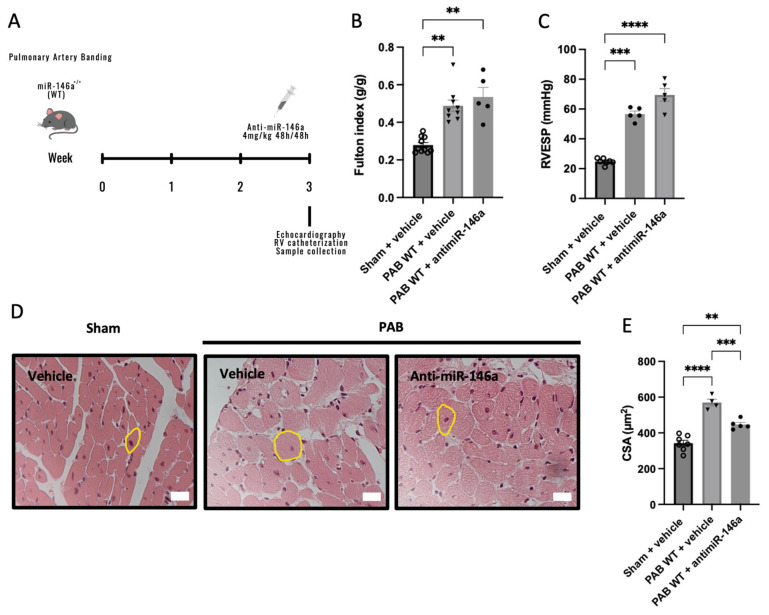
Wild-type (WT) mice were submitted to 3 weeks of pulmonary artery banding (PAB) and treated with miR-146a inhibitor (anti-miR-146a, 4 mg/kg) or negative control (vehicle) every 48 h for 1 week (**A**). RV hypertrophy, measured by the Fulton index (**B**), was increased in both PAB groups; however, the increase was more accentuated in the untreated PAB group compared with the treated PAB group. RV end-systolic pressures (RVESP) were increased in treated and untreated PAB mice compared with Sham animals (**C**). Cardiomyocyte hypertrophy, measured by cardiomyocyte cross-sectional area (CSA): representative cross-sections of cardiomyocytes (a yellow circle in representative cardiomyocytes) (**D**) and the quantitative analysis of cardiomyocyte CSA (**E**). Scale bar represents 50 μm in panel (**D**). Graph bars represent mean ± SEM for 4–10 mice. ** *p* < 0.005; *** *p* < 0.0005; **** *p* < 0.0001.

Beyond solely targeting the miR-146a gene, we also explored the impacts of therapeutic miR-146a inhibition in PAB mice. We administrated a miR-146a inhibitor (anti-miR-146a, 4 mg/kg) in PAB-WT mice (Figure 4A). PAB mice developed RV hypertrophy compared to Sham-WT mice, which was similar in PAB-vehicle and PAB-anti-miR146a mice (0.28 vs. 0.49 and 0.53 g/g, respectively) (Figure 4B). As expected, we found an increase in RVSP in PAB mice compared to Sham mice (56.64 and 69.48 vs. 24.76 mmHg, respectively) (Figure 4C). Regarding RV cardiomyocyte CSA, we found an increase in PAB-vehicle mice compared with Sham-WT and PAB-anti-miR-146a mice (569.58 vs. 342.57 and 446.89 µm^2^, respectively) (Figure 4D,E). There was an improvement in treated mice compared with untreated mice submitted to PAB in the RV cardiomyocyte hypertrophy (Figure 4E).

### 2.5. MiR-146a Inhibition Results in Significant Improvements in the Progression of PAH

**Figure 5:**
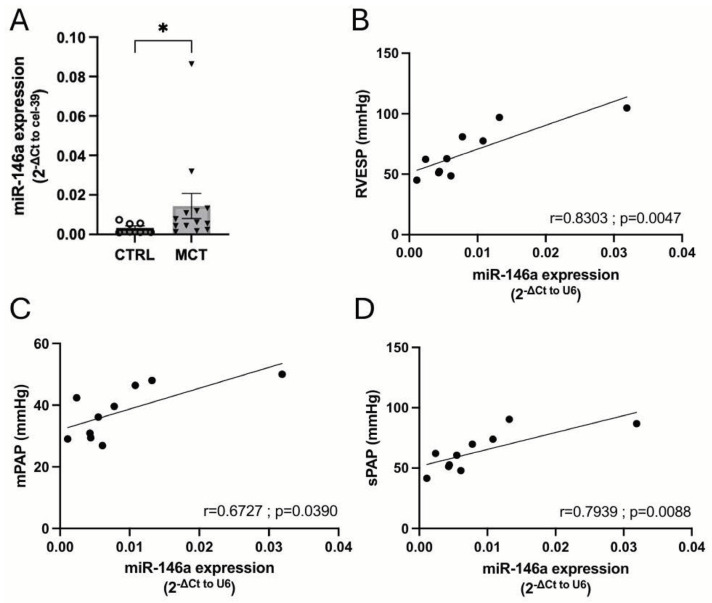
Plasmatic levels of miR-146a in control (CTRL) and monocrotaline (MCT) rats (**A**). Correlation between right ventricle systolic pressure (RVESP) (**B**), mean pulmonary arterial pressure (mPAP) (**C**), and systolic pulmonary arterial pressure (sPAP) (**D**) with the miR-146a expression on plasma in CTRL and MCT rats. Correlation was obtained by Spearman’s rank correlation. Graph bars represent mean ± SEM for 7–12 rats. * *p* < 0.05.

Finally, we analyzed a different animal model in rats, the monocrotaline (MCT) model. MCT-induced PAH has many similarities in terms of hemodynamic and histopathological severity with PAH in humans [29]. First, we evaluated the levels of miR-146a in the plasma of MCT-PH rats and found that plasmatic miR-146a levels were increased in MCT-PH rats compared to CTRL rats (Figure 5A). Interestingly, in CTRL and MCT-PH rats, the plasmatic miR-146a levels correlated to RVESP, mPAP, and sPAP (Figure 5B, Figure 5C and Figure 5D, respectively).

**Figure 6:**
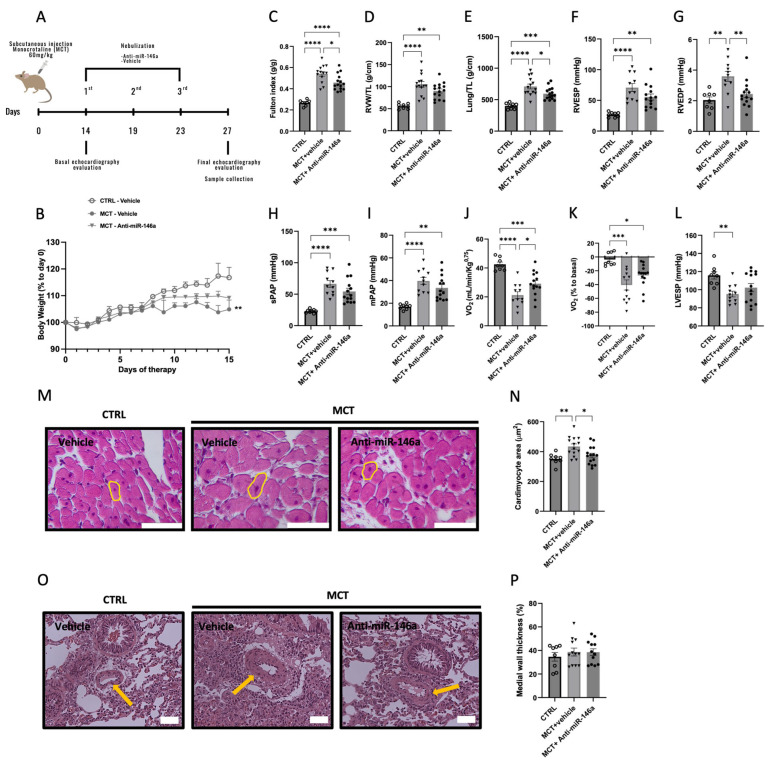
Sprague Dawley rats received a subcutaneous injection of 60 mg/Kg of monocrotaline (MCT) or vehicle (CTRL) and were treated with miR-146a inhibitor (anti-miR-146a, 2.5 nmol in 100 μL of sterile water or negative control (vehicle)) in three nebulizations (**A**). The percentage of body weight since the beginning of the protocol decreased in MCT untreated rats (**B**). Fulton index was increased in MCT groups compared with CTRL, with an improvement in MCT-treated rats (**C**). The weight of the right ventricle (RV) was increased in MCT groups (**D**). The weight of the lung was increased in MCT groups; however, this was more accentuated in MCT-untreated rats (**E**). RV end-systolic pressure (RVESP) was increased in MCT groups; however, a bigger increase was observed in MCT-untreated rats (**F**). RV end-diastolic pressure (RVEDP) was increased in the untreated MCT group, with an improvement in treated MCT rats similar to CTRL rats (**G**). Systolic and mean pulmonary artery pressures (sPAP and mPAP) were increased in MCT rats and more accentuated in untreated MCT rats ((**H**) and (**I**), respectively). The maximal oxygen consumption during the endurance test and the basal percentage were decreased in MCT untreated rats ((**J**) and (**K**), respectively). Left ventricle end-systolic pressure (LVESP) was decreased in MCT untreated rats (**L**). Cardiomyocyte hypertrophy, measured by cardiomyocyte cross-sectional area (CSA): representative cross-sections of cardiomyocytes (a yellow circle in representative cardiomyocytes) (**M**) and the quantitative analysis of cardiomyocyte CSA (**N**). Representative pulmonary arterioles in the lung sections of rats (a yellow arrow in representative pulmonary arterioles) (**O**) and the quantitative analysis of medial wall thicknesses (measured by inner diameter/(inner diameter + outside diameter) of pulmonary arterioles) (**P**). Scale bars represent 50 μm in panels (**M**,**O**). Graph bars represent mean ± SEM for 8–14 rats. * *p* < 0.05; ** *p* < 0.005; *** *p* < 0.0005; **** *p* < 0.0001.

Then, we administered miR-146a inhibitor (anti-miR-146a, 2.5 nmol in 100μl of sterile water) in vivo by nebulization from week 2 to week 3 to MCT-PH rats (Figure 6A). Regarding body weight, there was a significant decrease in MCT in untreated rats compared with the CTRL ones during the two weeks of treatment (Figure 6B). In the MCT-anti-miR-146a group, we found an improvement in RV hypertrophy (Fulton index, Figure 6C) and in RV end-diastolic pressure (RVEDP, Figure 6G) compared with the MCT-vehicle.

The weight of the RV and of the lung were increased in MCT groups compared to CTRL rats. However, the weight of the lungs was more accentuated in MCT-untreated rats than in MCT-treated rats (Figure 6E). This may be related to the accumulation of fluid in the lung, which could indicate the presence of pulmonary edema in MCT animals [30].

MCT-anti-miR146a rats presented an improvement in RV hypertrophy compared with MCT-vehicle rats, measured by the Fulton index (0.54 vs. 0.46 g/g) (Figure 6C) and lower systolic and diastolic pressures than MCT-vehicle rats (Figure 6F and Figure 6G, respectively). The treatment with anti-miR-146a decreased the RVEDP of treated MCT rats, similar to CTRL rats (2.44 vs. 2.04 mmHg, Figure 6G). sPAP and mPAP were increased in the MCT group, with an accentuated increase in MCT-vehicle rats compared to CTRL rats (66.28 vs. 22.98 and 39.73 vs. 16.99 mmHg, respectively; Figure 6H and Figure 6I, respectively). The maximal oxygen consumption during the endurance test and the basal percentage was lower in MCT-vehicle rats compared to MCT-anti-miR-146a rats (21.20 vs. 29.13 mL/min/Kg^0.75^ and −14.69 vs. −26.62% to basal, respectively; Figure 6J and Figure 6K, respectively). LV end-systolic pressure (LVESP) was decreased in MCT-vehicle rats compared to CTRL rats (95.19 vs. 115.78 mmHg, Figure 6L). The cardiomyocyte hypertrophy, measured by cardiomyocyte CSA, was increased in the MCT-vehicle group, and no differences existed between the CTRL and MCT-anti-miR-146a rats (434.62 vs. 351.97 and 374.43 µm^2^, respectively; Figure 6M,N). Regarding the medial thickness of the wall, no significant differences were observed between all experimental groups (Figure 6O,P).

**Figure 7:**
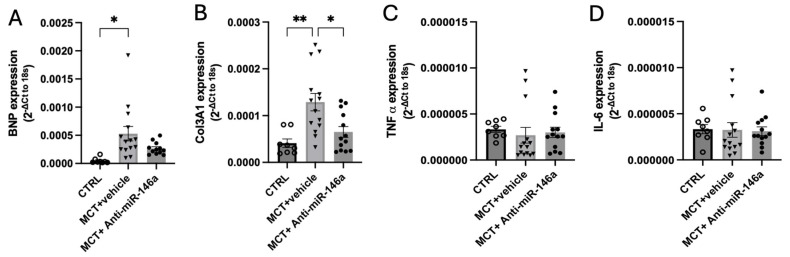
Cardiac effects of the inhibition of miR-146a by nebulization in monocrotaline (MCT) rats. Expression of cardiac dysfunction marker as brain natriuretic peptide (BNP) (**A**), a pro-fibrotic marker as COL3A1 (**B**), and inflammatory markers as TNF-α (**C**) and interleukin-6 (IL-6) (**D**) in the right ventricle (RV) of rats. Graph bars represent mean ± SEM for 8–14 rats. * *p* < 0.05; ** *p* < 0.005.

RV remodeling was associated with an increased mRNA expression of hypertrophic markers as brain natriuretic peptide (BNP) [31]. In MCT-exposed rats, BNP expression in the RV was increased (Figure 7A), indicating cardiac dysfunction. However, administering anti-miR-146a resulted in a noteworthy reduction in BNP levels among MCT rats, demonstrating the promising impact of anti-miR-146a treatment (Figure 7A). Additionally, compared to MCT-vehicle and CTRL rats, MCT-anti-miR-146a rats exhibited lower levels of COL3A1 (collagen-3), a pro-fibrotic marker involved in fibrotic vascular remodeling, highlighting the potential of anti-miR-146a therapy in mitigating fibrosis (Figure 7B). Despite the well-established RV inflammation in the MCT model contributing to RV dysfunction [32], we also analyzed the levels of two pro-inflammatory cytokines, TNF-α and interleukin-6 (IL-6). We did not find differences between TNF-α and IL-6 expression in all experimental groups (Figure 7C and Figure 7D, respectively).

### 2.6. Consequence of miR-146a Overexpression on hPASMCs

To understand the contribution of miR-146a overexpression to PAH pathogenesis, we evaluated the consequence of miR-146a overexpression on CTRL-hPASMCs. We evaluated the proliferation, migration, and apoptosis of CTRL-hPASMCs for 48 h after transfection with a mimic miR-146a (25 nM) and a mimic negative control (NC). Figure S2 shows the transfection efficacy with the mimic miR-146a (25 nM) in CTRL-hPASMCs, quantified by RT-PCR.

**Figure 8:**
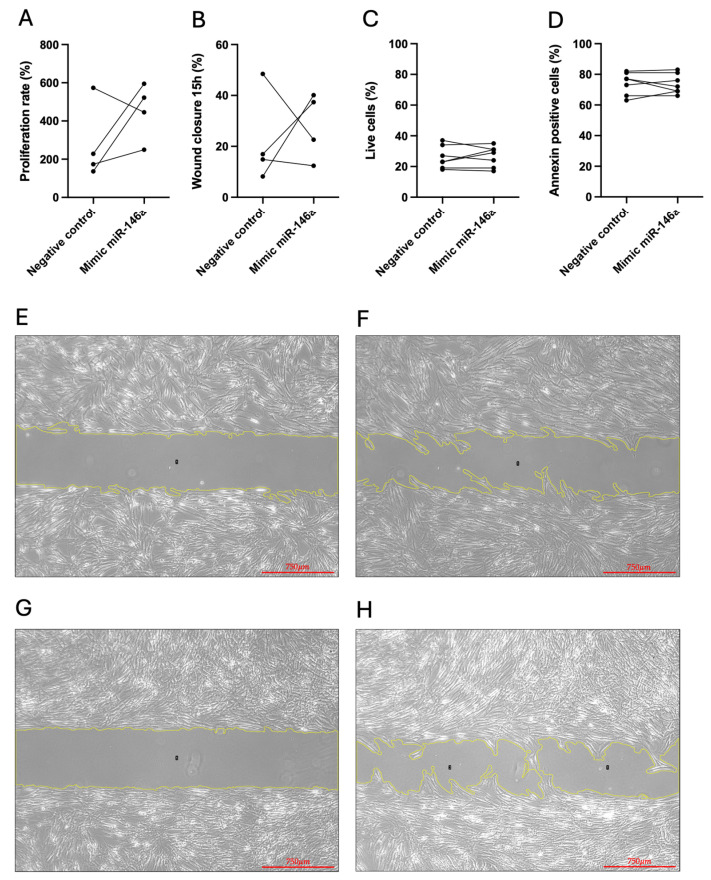
Effects of overexpression of miR-146a in control human pulmonary arterial smooth muscle cells (CTRL-hPASMCs) by transfection of a mimic miR-146a (25 nM) and a mimic negative control (NC). Consequences of miR-146a overexpression on the proliferation rate of CTRL-hPASMCs (**A**). Percentage wound closure 15 h after initiation in hPASMCs (**B**). The percentage of live cells on CTRL-hPASMCs resulting from miR-146a overexpression (**C**). The percentage of annexin-positive cells on CTRL-hPASMCs due to miR-146a overexpression (**D**). Wound closure images at time 0 h (**E**) and 15 h after (**F**) initiation of CTRL-hPASMCs transfected with mimic NC. Wound closure images at time 0 h (**G**) and 15 h after (**H**) initiation of CTRL-hPASMCs transfected with mimic miR-146a. Scale bars represent 750 μm in panels (**E**–**H**); 3–7 hPASMCs.

We found that miR-146a had no consequence on the proliferation rate (Figure 8A) and migratory capacity of CTRL-hPASMCs (Figure 8B,E–H), as well as in live cells and in apoptosis after staurosporine exposure (Figure 8C and Figure 8D, respectively). Figure S3 shows the flow cytometry plots of annexin V in CTRL-hPASMCs.

## 3. Discussion

PAH is a rare, severe form of PH with no effective treatment [33]. Research is urgently needed to better understand PAH and develop new therapies. This study investigates the role of miR-146a in PAH, emphasizing its positive effects on RV function.

We first show that miR-146a levels are increased in the lungs of PAH patients compared to CTRL ones (Figure 1A). Meanwhile, miR-146a expression shows no significant differences in hPASMCs and hPAECs between CTRL and PAH patients (Figure 1B and Figure 1C, respectively). The discrepancy between the increased miR-146a levels observed in the whole lung tissue of PAH patients and the lack of significant differences in miR-146a expression in isolated hPASMCs and hPAECs suggests a multifaceted explanation. The lung contains a heterogeneous population of cells (such as epithelial cells, immune cells, and connective tissue cells, as the fibroblasts), and the expression of miR-146a could vary significantly among different cell types. This small number of isolated cells (hPASMCs and hPAECs) might not capture the full spectrum of miR-146a expression changes occurring in the whole lung. Additionally, chronic inflammation associated with PAH involves immune cells known to express miR-146a, further contributing to the overall increase in lung tissue [34]. Moreover, activated fibroblasts involved in tissue remodeling and fibrosis in PAH [35] could also exhibit elevated miR-146a levels, an aspect not captured in hPASMC and hPAEC cultures.

We investigate the impact of miR-146a inhibition on PAH progression using animal models. Mice lacking miR-146a exposed to SuHx exhibit reduced RV hypertrophy (Figure 2). In an RV pressure overload model induced by PAB, KO mice showed improved RV hypertrophy and no RV dilation (Figure 3), indicating positive effects of miR-146a ablation on the RV. Additionally, PAB-WT mice treated with a miR-146a inhibitor showed improved RV hypertrophy regarding the cardiomyocyte CSA (Figure 4). The more significant impact of genetic ablation of miR-146a on the Fulton index and cardiomyocyte CSA compared to anti-miR-146a inhibition is likely due to the complete and permanent loss of miR-146a function, leading to more extensive physiological changes and the development of compensatory mechanisms. In contrast, using the anti-miR-146a provides only partial and transient inhibition, resulting in a less profound effect. Nebulization of a miR-146a inhibitor in MCT-PAH rats results in decreased RV hypertrophy and improved RV and PA pressures (Figure 6). Furthermore, MCT-anti-miR-146a rats show lower levels of the cardiac dysfunction markers BNP and COL3A1 compared to MCT-vehicle rats (Figure 7A and Figure 7B, respectively), confirming miR-146a’s involvement in PAH pathophysiology and supporting the potential therapeutic benefits of miR-146a inhibition in mitigating RV failure and PAH progression. Inhalation drug administration offers several advantages for treating PAH, including direct delivery to the lungs, enhancing pulmonary specificity, and minimizing systemic side effects. This method also improves ventilation/perfusion matching by dilating arteries in ventilated areas, enhancing gas exchange [36]. Nebulization is preferred for delivering substantial doses of inhaled medication during acute asthma, pneumonia, or chronic obstructive pulmonary disease episodes [37]. MCT-induced PH is associated with increased RV hypertrophy, medial thickness of the PAs, endothelial toxicity, and lung inflammation [38]. Our study showed that MCT-induced PH rats had significant RV hypertrophy, which improved with nebulized anti-miR-146a treatment. While the exact role of miR-146a and its inhibition is unclear, our findings suggest that nebulization with anti-miR-146a benefits RV function. Although the direct effects on pulmonary vasculature were minor, nebulized treatment may influence pulmonary dynamics, potentially causing vasodilation and reducing PAP, thereby improving RV function. Heuslein et al. demonstrated that knockdown of miR-146a reduces network-like formation and EC migration without affecting EC survival or permeability, indicating miR-146a’s role in vascular remodeling. Furthermore, miR-146a regulates perfusion recovery via arteriogenesis without significantly impacting angiogenesis. Targeting miR-146a could thus enhance vascular repair and improve blood flow in PAH patients. The lack of significant impact on angiogenesis is crucial, as it suggests therapeutic modulation of miR-146a can promote arterial remodeling and perfusion recovery without causing excessive capillary growth and related complications [39].

Recent studies have implicated miR-146a in cardiac conditions, including hypertrophy [40]. Our research shows that inhibiting miR-146a in animal models improves RV hypertrophy and dilation, enhancing cardiac function. MiR-146a-3p is upregulated in viral myocarditis, with cardiac progenitor cells releasing exosomes containing miR-146a-3p, highlighting its role in heart biology. Additionally, plasma levels of miR-146a-5p are significantly elevated in hypertrophic cardiomyopathy patients [40], indicating its potential as a novel marker for certain cardiovascular diseases.

MiR-146a LV expression increases in mice with pressure overload and in aortic stenosis patients [41]. Excessive miR-146a expression in cardiomyocytes induces hypertrophy and LV dysfunction, while its inhibition (genetic and pharmacological) mitigates these effects [41]. In sunitinib-induced cardiac dysfunction in mice, miR-146a overexpression protects against contractile dysfunction by modulating PLN (phospholamban) and ANK2 (ankyrin-B) expression [42]. Conversely, upregulated miR-146a in failing cardiomyocytes suppresses SUMO1 (small ubiquitin-like modifier 1), leading to contractile dysfunction, which is normalized by miR-146a inhibition [20]. MiR-146a is produced in fibroblasts and released in extracellular vesicles [20], suggesting distinct roles in various pathological conditions and effects on target gene expression.

In myocardial infarction (MI)-induced HF and cardiac remodeling in rats, inhibiting miR-146a improves LV systolic and diastolic pressures, while treatment with a miR-146a mimic worsens LVESP and LVEDP [43]. This aligns with our findings that miR-146a inhibition improves RV function. Additionally, miR-146a antagomiR inhibited the increase in levels of atrial natriuretic peptide (ANP), BNP, collagen I, and collagen III in the serum and heart of MI [43], consistent with our results, showing no difference in BNP expression and decreased collagen III expression in treated MCT rats. Thus, miR-146a inhibition may benefit both the right and left sides of the heart.

MiR-146a is crucial in immune responses, reducing cytokine production and hindering innate protection through TLR (Toll-like receptor) signaling [27]. It regulates inflammation by directly targeting TLRs and their downstream effectors, IRAK1 (interleukin-1 receptor-associated kinase 1) and TRAF6 (tumor necrosis factor receptor-associated factor 6) [44,45], and influences the transcription factor NF-kB (nuclear factor kappa B), forming a negative feedback loop for inflammation resolution [17,40,46]. Mice deficient in miR-146a expression exhibit excessive pro-inflammatory cytokine production in response to lipopolysaccharide (LPS), emphasizing miR-146a’s anti-inflammatory role [17]. Studies show that miR-146a regulates TRAF6 and IRAK1 expression, reducing inflammatory mediators like IL-6 and TNF-α [47,48,49]. Taking this into account, inhibition of miR-146a may be contradictory. However, in our study, we did not observe significant changes in the levels of the pro-inflammatory cytokines TNF-α and IL-6 (Figure 7C and Figure 7D, respectively) between treated and untreated MCT-induced PAH animals, suggesting miR-146a may not significantly regulate cytokines in this context. Supporting this, miR-146a inhibitors did not change IL-6 protein production in vitro [50]. Additionally, while MCT-exposed rats usually show increased IL-6 expression in the lung [51], our experiments do not show increased IL-6 expression in the RV, indicating no extra inflammation in the RV due to MCT.

In a pro-inflammatory environment, microvesicles from ECs increase smooth muscle cells (SMCs) proliferation and migration while decreasing apoptosis, linked with elevated miR-146a levels, suggesting miR-146a promotes SMC proliferation [52]. Studies have shown that miR-146a inhibition decreases vascular SMCs (VSMCs) proliferation and migration, while promoting VSMCs apoptosis [21,53]. Conversely, miR-146a overexpression promotes VSMC proliferation [53]. However, in our study, miR-146a overexpression does not affect hPASMC proliferation and migration as expected considering these previous studies, indicating that miR-146a’s pleiotropic effects may not influence the phenotypic characteristics of hPASMCs.

MiR-146a is involved in the increased production of extracellular matrix proteins, a key factor in several chronic diseases [26,54]. It regulates extracellular matrix protein production in diabetes and is upregulated in hypoxia-exposed hPAECs, suggesting a role in PAH [24,26,54]. MiR-146a also targets TRAF6 and inhibits NF-κB activity in ECs, reducing hypoxia-triggered inflammation and atherosclerosis. However, it promotes VSMCs and collagen deposition [55,56].

In humans, miR-146a regulates key genes in PAH pathogenesis such as BMPR2 (bone morphogenetic protein receptor type II), TGFBR2 (transforming growth factor-beta receptor 2), SMAD3, and SMAD5 [57]. Cold exposure exacerbates PAH in the MCT model by upregulating miR-146a in plasma and lungs, correlating with decreased expression of its targets. Elevated miR-146a levels inhibit SMAD3 expression, potentially altering TGFBR2 signaling. MiR-146a dysregulation is also implicated in acute lung injury [57]. In vitro, miR-146a-5p overexpression reduces SMAD3 expression, suppressing cell proliferation and promoting apoptosis, while its inhibition shows opposite effects [58]. Loss of SMAD3 enhances proliferation and migration in hPASMCs and hPAECs [59]. Although TargetScan identifies SMAD4 as a miR-146a target [58], our study found no differences in SMAD4 protein levels in PAH- and CTRL-hPASMCs upon miR-146a overexpression (Figure S4).

In the present study, miR-146a KO mice exhibited reduced RV remodeling and improved function following SuHx or PAB compared to WT mice. Pharmacological inhibition of miR-146a yielded a similar outcome, decreasing hypertrophy under pressure overload in PAB animals and enhancing cardiac function in MCT-exposed animals. Previous findings in experimental hypertensive HF suggest that miR-146a absence or inhibition attenuates cardiac hypertrophy and enhances function under LV pressure overload, whereas its overexpression exacerbates hypertrophy [19].

While miR-146a levels are elevated in the lungs of PAH patients compared to CTRL, no differences are observed in hPASMCs and hPAECs from PAH patients. Our findings indicate that miR-146a overexpression does not significantly impact proliferation, migration, or apoptosis in CTRL-hPASMCs. However, a potential limitation of our study is that CTRL patients’ cells are derived from individuals with lung cancer, which could potentially confound the results due to the frequent overexpression of miR-146a in malignant tumors [27]. Additionally, although the dose of mimic miR-146a used (25 nM) has been previously validated for its effects on miR-146a expression levels, it is possible that this dosage may not adequately modulate the signaling pathways regulated by miR-146a, thus potentially limiting its impact on proliferation, migration, and apoptosis in hPASMCs.

This study has several limitations. Firstly, the heterogeneity of the patient population, represented by variations in age, sex, genetic status, disease severity, and comorbidities, can complicate result interpretation and limit the generalizability of findings to the broader PAH patient population. Additionally, in vitro studies using human cells, like hPASMCs and hPAECs, may face limitations, including potential discrepancies in cellular representation, compared to the in vivo environment. Moreover, a limitation of nebulization in our study could be the loss of drug efficacy over the course of treatment, potentially reducing the effectiveness of the effects of anti-miR-146a in rats.

In conclusion, both genetic ablation and pharmacological inhibition of miR-146a show improvements in RV function and remodeling across various experimental models, indicating potential cardiac-specific actions of miR-146a in PAH. Nonetheless, elucidating the mechanisms through which miR-146a operates is crucial, and could offer valuable insights into PAH pathophysiology, potentially paving the way for innovative therapeutic strategies.

## 4. Materials and Methods

### 4.1. Patients and Human Lung Samples

Human lung specimens (specifically, lung parenchyma tissue) were obtained at lung transplantation in patients with PAH and lobectomy or pneumonectomy for localized lung cancer from patients as CTRL subjects. Around 70% of primary cells were derived from female PAH patients. PAs were isolated from away tumor areas in the lung specimens of control subjects. Transthoracic echocardiography was performed preoperatively in control subjects to rule out PH. Patients with PAH underwent genetic counseling and provided written informed consent for genetic analysis. The information on PAH and CTRL patients are presented in Table S1.

Patients were part of the French Network on PH, a program approved by the Université Paris-Saclay, Faculté de Médecine ethics committee, and provided written informed consent (Protocol N8CO-08-003, ID RCB: 2008-A00485-50, approved on 18 June 2008). All human tissues were obtained with written informed consent from transplant recipients or families of organ donors in accordance with the Declaration of Helsinki.

### 4.2. Animal Models

All animal experiments were performed in accordance with the recommendations of the Guide for the Care and Use of Laboratory Animals, published by the US National Institutes of Health (NIH Publication No. 85-23, Revised 2011), and were approved by the ethical committee of the Faculty of Medicine of the University of Porto and the Portuguese Foundation for Science and Technology (IMPAcT-PTDC/MED-FSL/31719/2017; POCI-01-0145-FEDER-031719), and certified by the Portuguese National Authority for Animal Health—DGAV (Direção-Geral de Alimentação e Veterinária) (0421/000/000/2013). All animal manipulations were executed by trained researchers who were certified with a Laboratory Animal Sciences course, according to the Federation of European Laboratory Animal Science Association. All animals were maintained in the specific pathogen-free zone of the animal facility at the Faculty of Medicine of the University of Porto. Animals were kept under a controlled environment with a 12 h light-dark cycle at 22 °C room temperature, with water and food ad libitum.

#### 4.2.1. Sugen 5416/Hypoxia-Induced Pulmonary Hypertension

Seven-week-old (18–20 g) male mice (WT and miR-146a^−/−^) were submitted to chronic hypoxia (10% O_2_) for 3 weeks, with weekly subcutaneous injections of Sugen 5416 (SU5416, 20 mg/kg, Tocris Biosciences, Ellisvile, MO, USA), to induce PAH (SuHx). The hypoxic chamber (BioSpheryx, Parish, NY, USA) was opened two times a week to clean animal cages and replenish food and water.

#### 4.2.2. Pulmonary Artery Banding (PAB)-Induced Right Ventricle Hypertrophy

Seven-week-old (18–20 g) male mice (WT and miR-146a^−/−^) were anesthetized (8% and 2.5–3% sevoflurane for induction and maintenance, respectively), intubated, and connected to a rodent ventilator (MouseVent G500, KentScientific, Torrington, CT, USA), with weight-defined tidal volume, controlled temperature and measured SpO_2_, heart rate, and end-tidal CO_2_. After depilating and disinfecting the left thorax, a small incision was made at the axillary level, the pectoral muscles were dissected and retracted, and an incision was made in the second intercostal space, exposing the PA. After carefully separating the PA from the left atria and aorta, a 7-0 prolene suture was passed around the PA and tied against a needle (23 gauge), resulting in a specified constriction. The thorax was closed, the pectoral muscles were put back in place, and the skin was sutured. Before the beginning of the protocol, the incision site was infiltrated with 1% lidocaine, and morphine (4 mg/kg) was administered subcutaneously before the procedure and every 4 h for the following 2 days. Sham animals underwent the same protocol, except that the suture around the PA was loosely tied. No post-surgical mortality was observed 3 weeks after PAB.

A subgroup of WT animals submitted to PAB was randomly assigned to receive an intravenous administration of anti-miR-146a (4 mg/kg, 100 μL, Thermo Fisher Scientific, Waltham, MA, USA) every 48 h during a week, according to previous works [60,61,62].

#### 4.2.3. Monocrotaline (MCT)-Induced Pulmonary Arterial Hypertension

Male Sprague Dawley rats weighing 150–200 g were purchased from Charles River Laboratories. Rats were randomly assigned to receive a single subcutaneous injection of 60 mg/kg of MCT (Sigma-Aldrich, St. Louis, MO, USA) or an equal volume of vehicle [63,64]. Fourteen days after the injection, the animals were randomly divided into 3 groups: a CTRL-vehicle, an MCT-vehicle, and an MCT-anti-miR-146a group. The MCT-anti-miR-146a group received intratracheal nebulization of anti-miR-146a (Ambion; 2.5 nmol in 100 μL of sterile water), and the other two groups received the vehicle. Rats were anesthetized with inhalation of 8% sevoflurane in a closed chamber for induction and 2–3.5% for maintenance and intubated orotracheally. Anti-miR-146a and the vehicle were nebulized three times (days 14, 19, and 23 after MCT injection) using an nebulizer (emka TECHNOLOGIES, Inc., Paris, France).

### 4.3. Echocardiography

Echocardiographic analysis was performed in male C57BL/6J mice 3 weeks after SuHx or PAB and in male Sprague Dawley rats 27 days after MCT administration, according to previous works [63,64]. Anesthetized (inhalation of 8% sevoflurane in a closed chamber for induction, and 2–3.5% for maintenance through a nose cone) and spontaneously breathing animals were placed in left lateral decubitus, on a heating pad with homeothermic control, through a rectal probe. The thorax was depilated, cleaned, and interrogated with a 14 MHz linear probe (15L8 probe coupled to an Acuson Sequoia C512 echocardiography system (Siemens Healthineers, Malvern, PA, USA) to evaluate RV structure and function.

### 4.4. Right Heart Catheterization

Following the echocardiographic evaluation, as described [61,64], animals were intubated and connected to a small animal ventilator, as mentioned before. The animal was positioned in right lateral decubitus, a left thoracotomy was performed, and a catheter was inserted through the apex of the RV and left ventricle (LV) positioned along the long axis (PVR-1035, Millar Instruments, Houston, TX, USA). Pressure signals were continuously acquired (MPVS Ultra, Millar Instruments, Houston, TX, USA), digitally recorded (PowerLab 16/30, ADInstruments, Dunedin, New Zealand), and analyzed offline (LabChart 7 Pro, ADInstruments). Following anesthetic overdose, the heart and lungs were removed en bloc, and the RV and LV + septum (LV + S) were carefully dissected, weighted separately, and collected for further analysis.

### 4.5. Histological Analysis

RV and lung samples were submerged in a fixative solution (10% formol), dehydrated, and submitted for diaphanization and inclusion in liquid paraffin. Four 4 μm sections were obtained and stained with hematoxylin-eosin to evaluate cardiomyocyte hypertrophy and pulmonary vascular remodeling. Sections were photographed (Olympus XC30) and analyzed offline (Cell^B, Olympus, Tokyo, Japan).

### 4.6. In Vitro

hPASMCs and hPAECs were cultured as previously described [65,66] and were used for the study between passages 3 and 5. hPASMCs were isolated from PAs obtained during lung transplantation for PAH patients and lobectomy or pneumonectomy of localized lung cancer from control subjects. PAs were excised at a distance from tumor areas.

#### 4.6.1. MiR-146a Transfection

hPASMCs were transfected in suspension by incubating 4 × 10^5^ cells in a solution containing 500 μL of Opti-MEM (Thermo Fisher Scientific, Waltham, MA, USA), 3 μL of Lipofectamine 1000 (Thermo Fisher Scientific, Waltham, MA, USA), and 25 nM of a specific mimic miR-146a (Thermo Fisher Scientific, Waltham, MA, USA), as well as a mimic negative control (NC) (Thermo Fisher Scientific, Waltham, MA, USA) according to the manufacturer’s protocol. All experiments were performed 72 h after the transfection.

#### 4.6.2. hPASMCs Proliferation Measurement

To evaluate cell proliferation, we quantified 5-bromo-20-deoxyuridine (BrdU) incorporation in cells undergoing DNA replication using the DELFIA cell proliferation kit (AD0200, PerkinElmer, Waltham, MA, USA), according to the manufacturer’s recommendations. hPASMCs were treated with mimic miR-146a or mimic NC. Forty-eight hours after cell synchronization (0% SVF), cell proliferation was induced for 24 h by a medium containing 10% of serum in the presence of 1 μmol/L BrdU for 24 h (PerkinElmer, Waltham, MA, USA) [67]. The fluorescence signal in the 96-well plate was read with a FlexStation 3 Microplate Reader (Molecular Devices, San Jose, CA, USA).

#### 4.6.3. Wound Healing Assay

To evaluate cell migration, we used a wound healing assay. After 48 h of starvation (medium without growth factors: FBS, EGF, and insulin), hPASMCs were plated in a culture insert (Cat. No. 90209; Ibidi GmbH, Gräfelfing, Germany) at a density of 1.2 × 10^4^ cells per well in a fresh medium with cytosine arabinoside (10 μmol/L). After allowing cells to attach for 24 h, we removed the culture insert and washed the cells with phosphate-buffered saline to remove non-adherent cells. Cells were treated with mimic miR-146a or mimic NC 24 h before starvation (72 h before removal of culture inserts). Cell migration into the wound space was quantified using ImageJ2. Cell motility/invasion was assessed by the percentage of wound closure 15 h after initiation of wound healing ([(area T_0_–area T_15_)/area T_15_] × 100) [68].

#### 4.6.4. hPASMCs Induced-Apoptosis Measurement

In hPASMCs, apoptosis was induced with 50 nmol/L staurosporine overnight. Cells were washed with cell-staining buffer (Cat. No. 420201, BioLegend, San Diego, CA, USA) and gently detached with trypsin and a cell scraper. After centrifugation (400× *g*, 4 °C, 10 min), the supernatant was removed, and cells were stained with the FITC Annexin V Apoptosis Detection Kit (Cat. No. 640914, BioLegend, San Diego, CA, USA) according to the manufacturer’s recommendations. Cells were acquired by flow cytometry (MACSQuant, Miltenyi Biotec B.V. & Co. KG, Bergisch Gladbach, Germany), with 75,000 cells counted in each sample and analyzed using FlowLogic^TM^ V.8 software (Inivai Technologies, Mentone, VIC, Australia).

### 4.7. Quantitative RT-PCR

Total RNA and miR were isolated from tissue and cells using the RNeasy mini kit according to the manufacturer’s instructions (Qiagen, Hilden, Germany). MiR was isolated from plasma samples using the miRNeasy mini kit according to the manufacturer’s instructions (Qiagen, Hiden, Germany). For miR quantification, a TaqMan^TM^ MicroRNA Assays (Applied Biosystems, Waltham, MA, USA) kit was used, a two-step protocol that requires only reverse transcription with an miRNA-specific primer, followed by real-time polymerase chain reaction (PCR) with TaqMan probes. For miR-146a, the assay ID: 000468 was used, and the results were normalized with U6 snRNA (assay ID: 001973, for tissue and cells) or with a spike-in cel-miR-39 (assay ID: 000200, for plasma). For the reverse transcription reaction, a TaqMan^TM^ MicroRNA reverse transcription kit (Applied Biosystems, Waltham, MA, USA) was used, and for the PCR reaction, a TaqMan^TM^ Universal PCR Master Mix (Applied Biosystems, Waltham, MA, USA) was used, following the manufacturer’s instructions. For mRNA quantification, cDNA was synthesized using SuperScript II reverse transcriptase (Invitrogen, Waltham, MA, USA), and the PCR was performed with a SYBR Green PCR master mix (Applied Biosystems, Waltham, MA, USA), normalized for 18 s. The specific primer sequences can be found in Table S2. qRT-PCR was performed with the ABI Prism 7900 Sequence Detector (Applied Biosystems, Waltham, MA, USA). The relative expression level was determined by the 2^−ΔCt^ method.

### 4.8. Statistical Analysis

Statistical analysis was performed using GraphPad Software (v10). After checking the distribution of our samples, differences between two or more groups were assessed using one-way or two-way ANOVA, followed by Tukey’s analysis or Šídák’s analysis for multiple comparisons, respectively. When conditions for parametric tests were not found, differences between the two groups were analyzed using the Mann–Whitney test or the Wilcoxon matched-pairs signed rank test. All data were presented as mean ± SEM. The correlation between miR-146a expression in plasma and hemodynamic parameters was obtained by Spearman’s rank correlation. Differences with *p* < 0.05 were considered statistically significant for all experiments.

## Data Availability

The data presented in this study are available on request from the corresponding author.

## Supplementary Materials

The following supporting information can be downloaded at: https://www.mdpi.com/article/10.3390/ijms25158054/s1.
