# Supplementary material for: Unraveling the Impact of miR-146a in Pulmonary Arterial Hypertension Pathophysiology and Right Ventricular Function
Source: Int J Mol Sci. 2024 Jul 24;25(15):8054. doi: 10.3390/ijms25158054 (PMC11311781; doi:10.3390/ijms25158054)
Supplement: Supplementary file 1 [file ijms-25-08054-s001.zip › ijms-3095897-supplementary.pdf]

Supplementary File

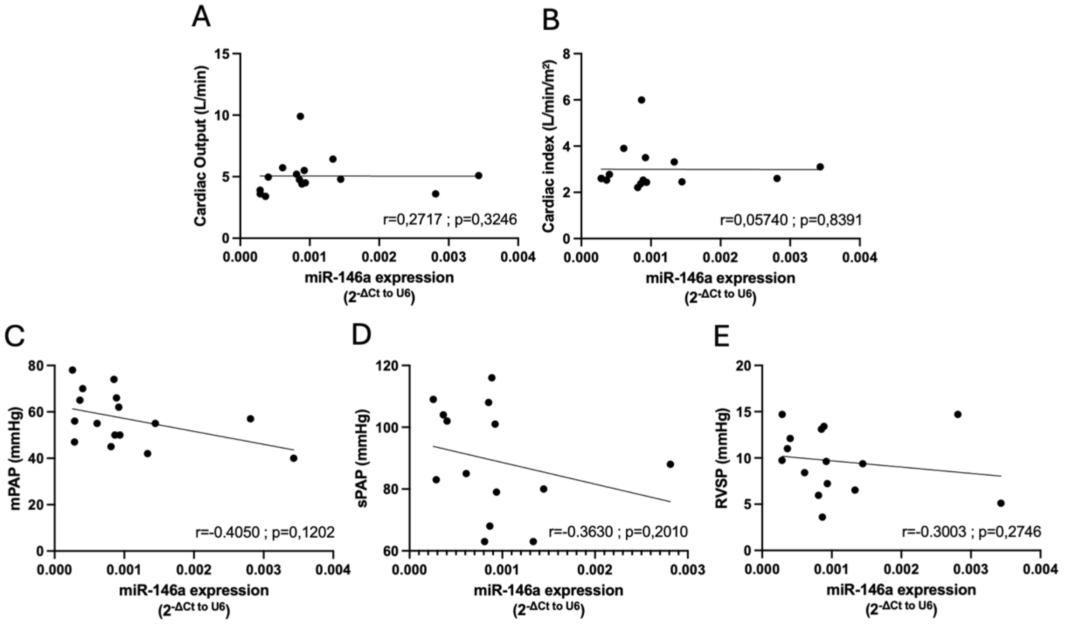

Figure S1- Correlation between cardiac output (A), cardiac index (B), mean pulmonary arterial pressure (mPAP) (C), systolic pulmonary arterial pressure (sPAP) (D) and right ventricle systolic pressure (RVSP) (E) with the miR-146a expression on lung tissue in pulmonary arterial hypertension (PAH) patients. Correlation was obtained by Spearman's rank Correlation (n=20).

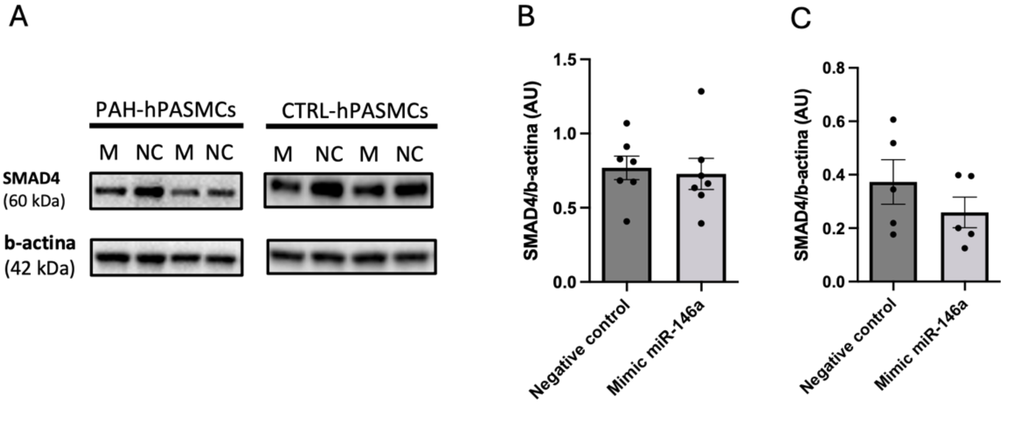

Figure S2- Protein expression of Smad4. Representative blots of SMAD4 in pulmonary arterial hypertension (PAH)- human pulmonary arterial smooth muscle cells (PAH-hPASMCs) and control-hPASMCs (CTRL-hPASMCs) when transfected with mimic-miR-146a (M) and negative control (NC) (A). SMAD4 protein levels in PAH-hPASMCs (B) and in CTRL-hPASMCs (C). Graph bars represent mean ± SEM. 5-7 hPASMCs. AU- arbitrary units.

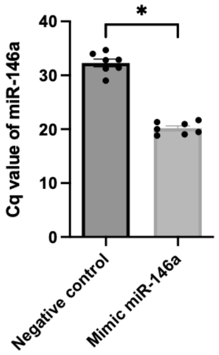

Figure S3- Cq value of miR-146a in control (CTRL) human pulmonary arterial smooth muscle cells (hPASMCS) transfected with mimic miR-146a with a dosis of 25nM and negative control. Graph bars represent mean ± SEM. (n=7) *p<0.05

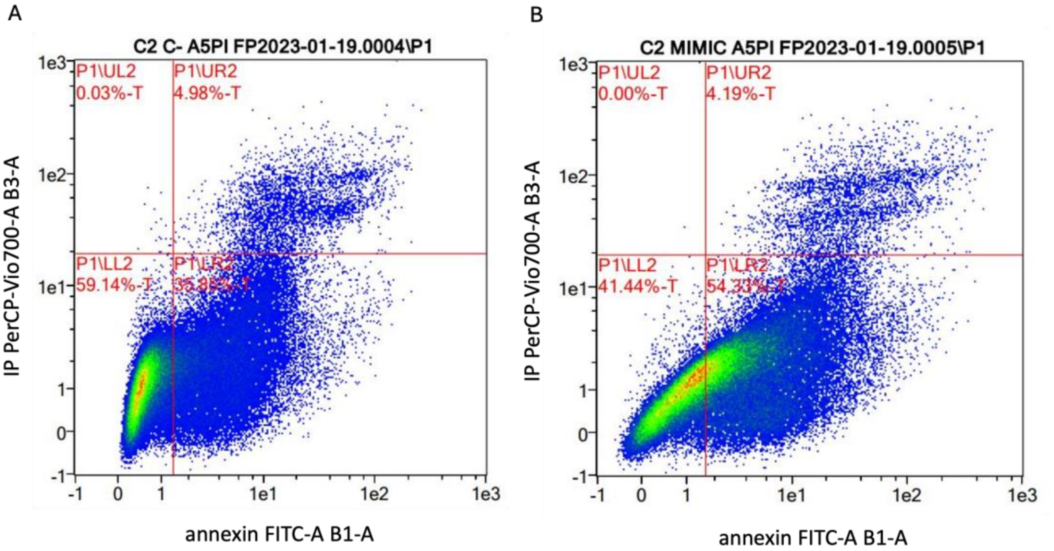

Figure S4- Flow cytometry plots for the annexin V in CTRL-hPASMCs transfected with NC (A) and mimic miR-146a (B).

Table S1 –Parameters from pulmonary arterial hypertension (PAH) patients examined alongside miR-146a levels in lung tissue. sPAP- systolic pulmonary arterial pressure; dPAP- diastolic pulmonary arterial pressure; mPAP- mean pulmonary arterial pressure; RVP- right ventricle pressure (n=20).

Patient	Age	Sex	sPAP (mmHg)	dPAP (mmHg)	mPAP (mmHg)	Cardiac Output (L/min)	RVP (mmHg)	Right atrium pressure (mmHg)	Cardiac Index (L/min/m^2)	Pwedge pressure (mmHg)	miR-146a expression (2^(-ΔCt to U6))
PAH 1	47	F	104	38	65	3,4	11	9	2,53	17	0,0003641
PAH 2	44	F	120								0,0032471
PAH 3	49	F									0,0002739
PAH 4	25	F									0,0006309
PAH 5	48	F	79	45	50	4,5	7,22	9	2,44	17	0,0009374
PAH 6	53	M	63	37	45	5,2	5,96	21	2,21	14	0,0008096
PAH 7	49	M			40	5,1	5,1	5	3,1	6	0,0034314
PAH 8	36	M	108	49	74	4,76	13,1	8	2,37	12	0,0008526
PAH 9	48	M	63	30	42	6,43	6,53	19	3,32	11	0,0013350
PAH 10	29	F	68	24	50	9,9	3,6	17	6	14	0,0008658
PAH 11	28	F	116	36	66	4,4	13,4	4	2,53	7	0,0008881
PAH 12	50	M	109	54	78			9		9	0,0002566
PAH 13	32	F	80	43	55	4,8	9,38	10	2,46	10	0,0014456
PAH 14	46	F	88	37	57	3,6	14,7	2	2,6	4	0,0028127
PAH 15	37	F			47	3,9	9,74	10	2,6	9	0,0002858
PAH 16	52	F	83	31	56	3,6	14,7	2	2,6	4	0,0002880
PAH 17	45	M									0,0003023
PAH 18	23	M	102	46	70	4,97	12,1	9	2,78	10	0,0004035
PAH 19	49	49	85	35	55	5,73	8,4	2	3,9	7	0,0006098
PAH 20	31	31	101	41	62	5,5	9,63	18	3,5	8	0,0009216

Table S2 –Sequence of primers used.

Name	Sequence (5`- 3`)
RnIL-6_F1<br>RnIL-6_R1	ATCTGCTCTGGTCTTCTGGA<br>TTGCTCTGAATGACTCTGGCT
RnTNFalpha_F1<br>RnTNFalpha_R1	CCACCACGCTCTTCTGTCT<br>CTACGGGCTTGTCACTCG
Rn18s_F1<br>Rn18s_R1	CGTCTGCCCTATCAACTTTCG<br>CTTGGATGTGGTAGCCGTTT
RnNppb_F1<br>RnNppb_R1	CTGTCGCCGCTGGGAGGTCACT<br>AGCCATTTCCTCTGACTTTTCTC
RnCol3a1_F1<br>RnCol3a1_R1	ATATCAAACACGCAAGGC<br>GATTAAAGCAAGAGGAACAC
